# Assessing Fibrosis Progression and Endothelial Dysfunction in SSc-ILD and COPD: An Integrated Biomarker and CT Densitometry Approach

**DOI:** 10.3390/medicina61091572

**Published:** 2025-08-31

**Authors:** Lyazat Ibrayeva, Irina Bacheva, Assel Alina, Olga Klassen

**Affiliations:** Department of Internal Medicine, Karaganda Medical University, Karaganda 100012, Kazakhstan

**Keywords:** COPD, SSc-ILD, endothelin-1, pulmonary fibrosis, lung–kidney interaction, organ crosstalk, biomarkers, galectin-3, quantitative densitometry

## Abstract

*Background and Objectives:* Chronic lung diseases act as multi-organ conditions in which systemic inflammation, vascular dysfunction, and fibrosis intersect. The pulmo-renal continuum—functional crosstalk between lungs and kidneys—remains poorly characterized. We compared year-long changes in endothelin-1 (ET-1), galectin-3 (Gal-3), renal indices (eGFR, ACR), and quantitative CT densitometry in COPD and systemic sclerosis-associated ILD (SSc-ILD). *Materials and Methods*: In this prospective observational study (January 2023–December 2024), 112 patients were consecutively enrolled (COPD, n = 58; SSc-ILD, n = 54). Assessments were performed at baseline and 12 months. ET-1 (ELISA) and Gal-3 (chemiluminescence) were measured in serum; eGFR was calculated by the creatinine-based CKD-EPI (2021) equation; ACR was photometric. High-resolution chest CT provided lung volume and parenchymal density (Hounsfield units) at six predefined axial levels per lung. Non-parametric statistics were applied: Wilcoxon signed-rank (within-group), Mann–Whitney U (between-group), and Spearman rank correlations for associations; results are reported with *p*-values (and 95% CIs). *Results:* Baseline eGFR was normal (COPD 90.37; SSc-ILD 92.4 mL/min/1.73 m^2^). eGFR declined by 6.76% in COPD (*p* = 0.001) and 3.16% in SSc-ILD (*p* = 0.029). ET-1 increased in both cohorts but more in COPD (+83.78%, *p* = 0.0002) than in SSc-ILD (+23.83%, *p* = 0.0001). Gal-3 rose significantly only in SSc-ILD (+10.2%, *p* = 0.043). FVC decreased in COPD (−4.01%, *p* = 0.01) and was unchanged in SSc-ILD. Total lung volume declined in SSc-ILD (−6.08%, *p* = 0.02) but not in COPD. CT density shifts were small: several slices in COPD and one slice (L6) in SSc-ILD reached statistical but not biological relevance. *Conclusions:* COPD exhibited larger vascular and renal biomarker shifts (ET-1 up, eGFR down, ACR up), suggesting systemic inflammation and early renal involvement. In SSc-ILD, biomarker and CT changes predominantly reflected pulmonary fibrosis progression with limited renal impact. Integrating biomarkers with quantitative CT may help delineate organ-specific trajectories along the pulmo-renal continuum; longer, larger studies are warranted. Limitations: This was a single-center cohort with a modest sample (58 COPD and 54 SSc-ILD) and a 12-month, two-time-point follow-up, which may not capture long-term trajectories and may limit it generalizability; larger multicenter studies with an extended follow-up are warranted.

## 1. Introduction

Chronic lung diseases are increasingly regarded not as isolated respiratory disorders but as multi-organ conditions driven by systemic inflammation, vascular dysfunction, and fibrosis-induced remodeling processes [1,2]. One of the least explored yet clinically relevant manifestations of this systemic nature is the pulmo-renal continuum—bidirectional functional and biochemical interactions between the lungs and kidneys. In this continuum, pathological processes in one organ can trigger or exacerbate dysfunction in the other through shared pathways such as systemic inflammation, endothelial injury, oxidative stress, and neurohormonal activation. This link means that chronic lung disease may contribute to gradual renal impairment, while kidney dysfunction can worsen pulmonary outcomes [2,3,4,5,6]. Large epidemiological studies indicate that chronic obstructive pulmonary disease (COPD) increases the risk of a reduced estimated glomerular filtration rate (eGFR) by 1.5–2-fold, whereas co-existing chronic kidney disease (CKD) worsens outcomes in patients with interstitial lung disease (ILD) [7,8].

A pivotal axis of lung–kidney crosstalk is the endothelin system. Endothelin-1 (ET-1) is a potent vasoconstrictor, a mediator of endothelial dysfunction, and a driver of fibrogenesis [9,10,11,12]. ET-1 levels rise during COPD exacerbations and with progression of systemic sclerosis-associated ILD (SSc-ILD), yet comparative longitudinal data on ET-1 dynamics across these entities remain scarce. Galectin-3 (Gal-3), a fibrosis-related biomarker involved in macrophage activation and matrix remodeling, has been linked to faster lung function decline, radiographic fibrosis progression, and increased mortality in systemic sclerosis-associated pulmonary fibrosis [13].

Contemporary computed tomography (CT) techniques permit quantitative assessment of parenchymal fibrosis via densitometry expressed in Hounsfield units (HU). Integrating serial CT densitometry with measurements of vascular–fibrotic biomarkers provides a multimodal framework for monitoring fibrotic activity and endothelial dysfunction. However, to our knowledge, integrated year-long analyses of ET-1, Gal-3, renal indices (eGFR, ACR), and quantitative CT density in COPD and SSc-ILD remain scarce.

Recent longitudinal studies have demonstrated that key parameters can change within 1–2 years in both COPD and SSc-ILD. In COPD, this includes increases in ET-1 and Gal-3 during exacerbations, gradual decline in eGFR, rising albuminuria, and CT-documented progression of emphysema [14,15,16,17,18]. In SSc-ILD, HRCT is central for diagnosis, severity stratification, and monitoring; moreover, changes in extent of semi-quantitatively assessed ILD (ΔSQCT) over 12–24 months correlate significantly with a decline in lung function (FVC and DLco) [19,20]. In this group, eGFR generally remains stable, whereas Gal-3 levels are higher in patients with active disease [21,22]. However, evidence specifically addressing one-year longitudinal dynamics of ET-1 and Gal-3 in SSc-ILD remains scarce, representing an important gap in the literature.

We conducted a prospective one-year study with two assessment points in COPD and SSc-ILD cohorts. The aims were to compare the dynamics of ET-1 and Gal-3, assess renal function (eGFR, ACR), and evaluate quantitative CT metrics, as well as to explore their interrelationships within the pulmo-renal continuum. We hypothesized that in SSc-ILD, biomarker changes would primarily reflect fibrotic progression, whereas in COPD they would indicate systemic inflammation and early renal involvement. Through these analyses, we sought to refine the roles of ET-1 and Gal-3 as potential differential indicators and to provide a basis for future research on organ-specific fibrosis.

## 2. Materials and Methods

### 2.1. Study Population

**Figure 1:**
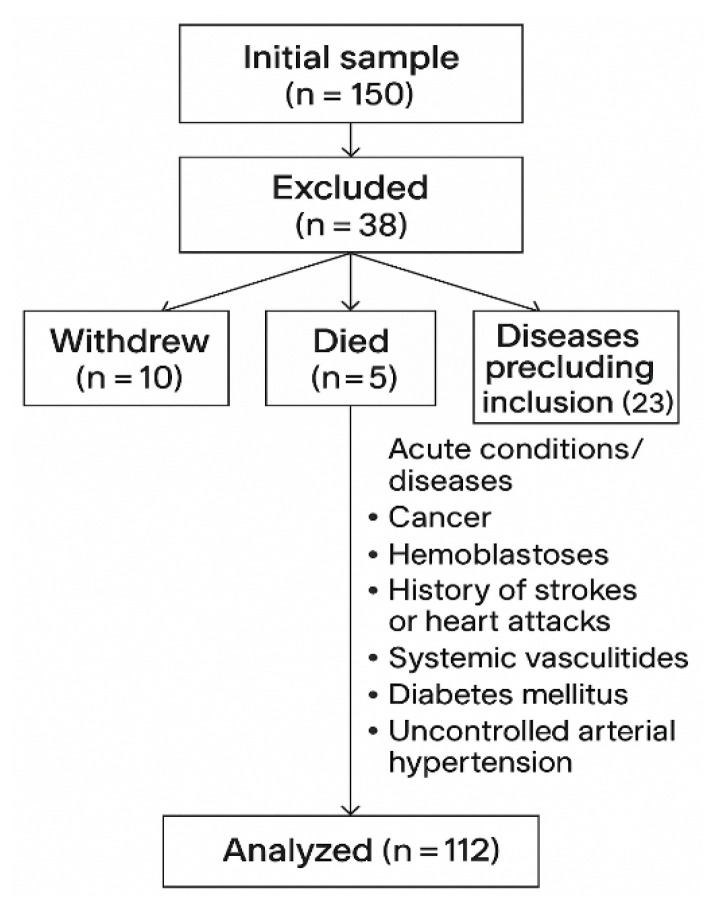
Flowchart of patient selection and reasons for exclusion.

This prospective observational study was conducted between January 2023 and December 2024. A total of 112 patients with confirmed chronic lung diseases, including chronic obstructive pulmonary disease (COPD, n = 58) systemic sclerosis-associated ILD (SSc-ILD, n = 54), were consecutively enrolled, and all 112 were analyzed. The participant flow diagram is presented in Figure 1.

Among the  58 COPD patients, 22 (38%) met GOLD stage II and 36 (62%) met stage III criteria. All COPD subjects had a smoking history > 10 pack-years. The SSc-ILD cohort (n = 54) consisted exclusively of systemic sclerosis-associated ILD (SSc-ILD). All participants were consecutively enrolled at the Regional Clinical Hospital (Karaganda, Kazakhstan).

### 2.2. Exclusion Criteria

Age < 25 or >65 years was an exclusion criterion, as were chronic heart failure with reduced or mildly reduced ejection fraction; pregnancy; baseline eGFR < 60 mL/min/1.73 m^2^; acute infections or exacerbations; solid or hematological malignancy; prior stroke or myocardial infarction; systemic vasculitis; diabetes mellitus; uncontrolled hypertension. Patients with baseline eGFR < 60 mL/min/1.73 m^2^ were excluded to focus on early renal changes rather than established chronic kidney disease. Patients with overlapping diagnoses of COPD and ILD were not included; only individuals with an isolated diagnosis of either condition were enrolled.

### 2.3. Imaging Protocol

High-resolution chest CT was acquired on a 160-slice scanner (Aquilion PRIME SP, Canon Medical Systems, Otawara, Tochigi, Japan; reconstruction kernel B70f, slice thickness 1 mm, 120 kV, automatic mA modulation). Quantitative lung densitometry was performed on six predefined axial levels per lung (Table 1) using VIDAR DICOM Viewer v3.0 (VIDAR Systems Corp., Herndon, VA, USA).; density was expressed in Hounsfield units (HU). For quantitative HRCT analysis, clinical and laboratory data were not available to the readers in order to minimize bias.

Patients were categorized into two subgroups by diagnosis (COPD vs. ILD).

### 2.4. Biomarker Analysis

Serum endothelin-1 was measured by competitive ELISA (Cloud-Clone Corp., Houston, TX, USA, CEA482Hu; 2 h assay; range 6.17–500 pg/mL; LoD < 2.7 pg/mL), performed in the Scientific Research Laboratory of Karaganda Medical University on an EVOLIS (Bio-Rad Laboratories, Inc., Hercules, CA, USA) automated analyzer. Serum creatinine and ACR were assessed photometrically. Galectin-3 (ng/mL) was measured by chemiluminescent assay in the accredited OLYMP laboratory (OLYMP, Karaganda, Kazakhstan; ISO 15189:2015) [23]. Renal function was evaluated using serum creatinine and the estimated GFR calculated via the creatinine-based CKD-EPI equation (2021) [24].

### 2.5. Statistical Analysis

All computations were performed in R v 4.3.2 (R Foundation for Statistical Computing, Vienna, Austria). Raw data were imported with readxl and processed with dplyr and tidyr (tidyverse collection). The he distribution of each variable was examined using the Shapiro–Wilk test. As none showed normality (*p* < 0.05), non-parametric statistics were applied. Paired observations from 2023 to 2024 within each group were compared with the two-sided Wilcoxon signed-rank test. Between-group differences were assessed with the Mann–Whitney U test. Associations between continuous variables were evaluated using Spearman rank correlation coefficients. Logarithmic change was calculated as log_2_FC = log_2_(value2023/value2024).

Volcano plots were produced with ggplot2 and ggrepel (x log_2_FC; y: −log_10_*p*). Statistical significance was set at *p* < 0.05. Negative log_2_FC values denote a decrease at follow-up.

A change was considered biologically relevant when |log_2_FC| > 0.20, which corresponds to a ≥15% relative difference (log_2_ 1.15 ≈ 0.20). This threshold exceeds both the analytical imprecision of the assays (<8% CV) and the expected day-to-day biological variation (<10%), ensuring that only changes of potential clinical importance are highlighted.

### 2.6. Ethical Approval

This study was approved by the Local Ethics Committee of Karaganda Medical University (Protocol No. 2, 11 October 2022) prior to initiation of the study. The observational phase ran from January 2023 to December 2024. All participants provided written informed consent. Patient data were anonymized and de-identified for analysis.

## 3. Results

### 3.1. Baseline Characteristics of the Groups

Of the 112 patients, 55.4% (62) were male and 44.6% (50) were female. The study included 58 COPD and 54 SSc-ILD patients. The median age of the participants was 54.5 [52.1–55.1]. The smoking status of the participants was as follows: 45.5% (51) were never smokers, 28.9% (22) were former smokers, and 51.3% (39) were current smokers. Demographic and spirometry data are presented in Table 2.

### 3.2. One-Year Dynamics of Renal Function, Biomarkers, Pulmonary Function, and CT Density in COPD Versus SSc-ILD

#### 3.2.1. Renal Function

Estimated GFR declined in both cohorts and reached statistical significance: −6.76% in COPD (*p* = 0.001) and −3.16% in SSc-ILD (*p* = 0.029). The albumin-to-creatinine ratio showed no biologically relevant change in either group (Table 3).

#### 3.2.2. Endothelin-1

Serum endothelin-1 increased both biologically and statistically in the two cohorts, but the increase was more pronounced in COPD (+83.78%, *p* = 0.0002) than in SSc-ILD (+23.83%, *p* = 0.0001).

#### 3.2.3. Galectin-3

A statistically significant elevation was observed only in SSc-ILD (+10.2%, *p* = 0.043); the magnitude approached, but did not exceed, the predefined threshold for biological relevance.

#### 3.2.4. Pulmonary Function

Forced vital capacity changed significantly in COPD (*p* = 0.01), yet the effect size was biologically trivial (−4.01%).

#### 3.2.5. CT Densitometry

Total lung volume decreased significantly in SSc-ILD (−6.08%, *p* = 0.02), but the reduction did not reach biological relevance; COPD patients showed a non-significant upward trend (+2.95%, *p* = 0.146). In COPD, percentage changes in parenchymal density did not exceed the predefined threshold for biological relevance, but several slices exhibited statistically significant shifts: mild densification in R1 (+0.07%, *p*= 0.001), L1 (+0.21%, *p* = 0.004) and R2 (+0.01%, *p* = 0.002), and slight rarefaction in R4 (−0.35%, *p* = 0.016). In SSc-ILD, only slice L6 showed a statistically significant change (+0.13%, *p* = 0.004); the magnitude was small and below the biological threshold.

#### 3.2.6. Functional Outcomes over One Year

Over one year, COPD patients remained stable, with no significant change in post-6MWT oxygen saturation (−1.1%, *p* = 0.128) and Borg dyspnea scores (+14.3%, *p* = 0.671). In contrast, SSc-ILD patients showed clear deterioration: post-6MWT saturation declined by −1.6% (*p* = 0.015), and Borg dyspnea scores increased by +33.3% (*p* = 0.002). Thus, unlike COPD, SSc-ILD demonstrated both objective (greater desaturation) and subjective (higher Borg scores) evidence of progressive respiratory impairment.

**Figure 2:**
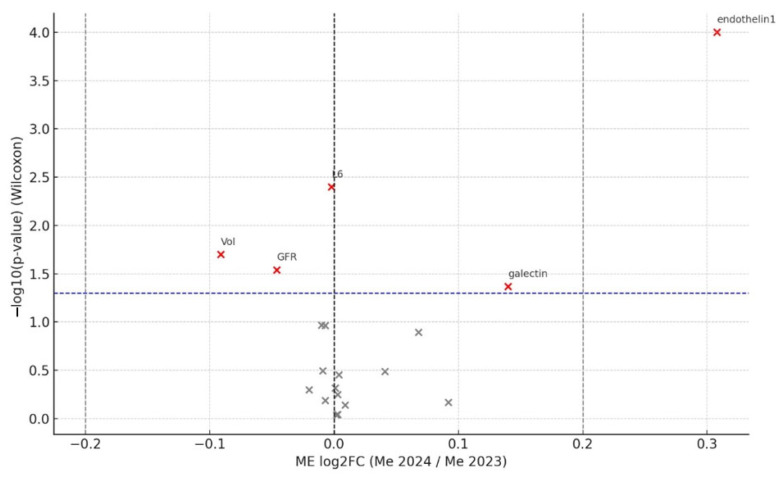
Volcano plot of within-cohort changes in the SSc-ILD group (2024 vs. 2023). The x-axis shows the median log_2_ fold change (log_2_FC = log_2_[2024/2023]); the y-axis shows −log_10_(*p*) from the Wilcoxon signed-rank test. Vertical dashed lines mark the biological relevance threshold (|log_2_FC| = 0.20), and the horizontal dashed line marks statistical significance (*p* = 0.05). Red points indicate variables meeting both criteria (statistical and biological); gray points do not. Abbreviations: ET-1, endothelin-1; Gal-3, galectin-3; eGFR, estimated glomerular filtration rate; ACR, albumin-to-creatinine ratio; FEV1, forced expiratory volume in 1 s; FVC, forced vital capacity; HU, Hounsfield units.

**Figure 3:**
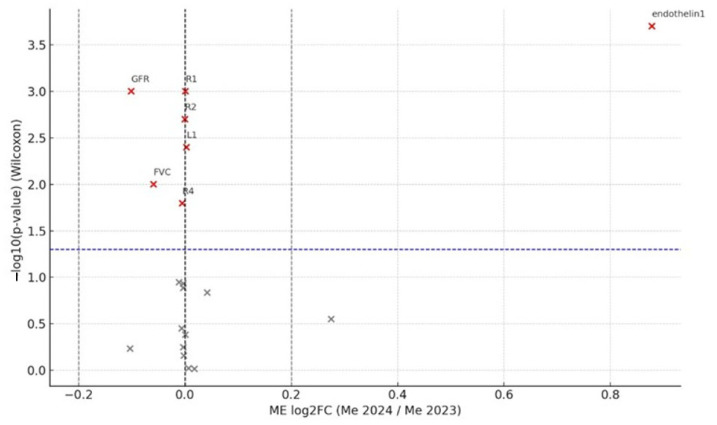
Volcano plot of within-cohort changes in the COPD group (2024 vs. 2023). Axes, thresholds, and color coding are the same as in Figure 2. ET-1 is the only marker exceeding both statistical and biological cut-offs. Abbreviations as in Figure 2.

Figure 2 and Figure 3 display volcano plots for COPD and SSc-ILD, respectively, highlighting biologically meaningful deviations.

Thus, in both cohorts only endothelin-1 demonstrated changes that were both statistically and biologically significant. In COPD patients, the eGFR and FVC decreased significantly by 7% and 4%, respectively, whereas CT-based parenchymal density changes in individual slices (R1, L1, R2, R4) were small—ranging from 0.07% to 0.35% over one year—and differed in direction (some densification, some rarefaction). In the SSc-ILD group, eGFR, total lung volume, and galectin-3 all reached statistical significance but not biological relevance: eGFR and lung volume fell by 3% and 6%, respectively, while galectin-3 increased by 10%.

### 3.3. Between-Group Comparisons (COPD vs. SSc-ILD)

Table 4 presents a comparison of the median parameter values between the COPD and ILD cohorts.

Between-group analysis showed that only the median changes in FVC and slice L2 were both statistically and biologically significant between the *SSc-ILD* and COPD cohorts. Biologically meaningful differences (Δdiff > 1 unit) were also observed for the following medians: endothelin-1 (−5.9), galectin-3 (+1.6), total lung volume (−24.5), L1 (+1.5), L2 (−2.0), R3 (−2.0), R4 (−1.5), R5 (−2.0), L5 (+1.5), and R6 (+2.0).

**Figure 4:**
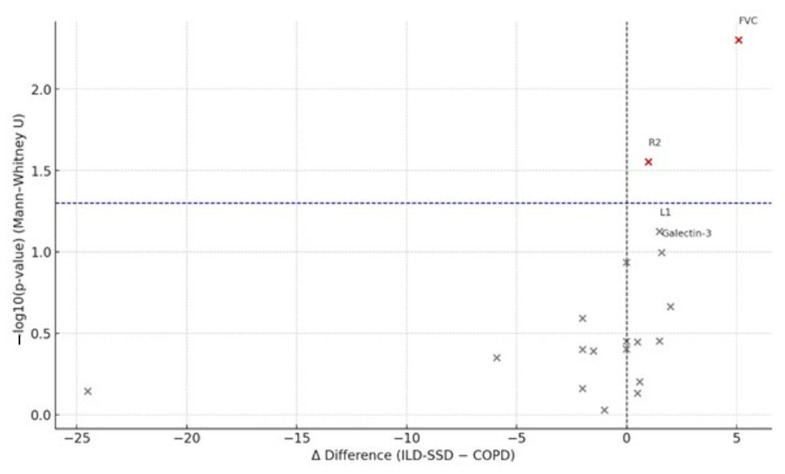
Volcano plot of between-group differences in median changes (Δ, SSc-ILD − COPD). The x-axis displays the Δ-median; the y-axis shows −log_10_(*p*) from the Mann–Whitney U test. The vertical dotted lines indicate the predefined threshold for biological relevance (|Δ| > 1 unit for illustrative purposes), and the horizontal dashed line indicates *p* = 0.05. Abbreviations as in Figure 2.

Figure 4 presents volcano plots of the Δ-median differences between the *SSc-ILD* and COPD groups, highlighting parameters with biologically significant deviations.

The median FVC showed differences that were both statistically and biologically significant, whereas the median density for slice R2 differed only statistically. The median galectin-3 level was biologically higher in the COPD group, but this change only approached statistical significance (*p* = 0.101). All other annual (Δ) changes in markers displayed neither biological nor statistical differences between the SSc-ILD and COPD cohorts.

### 3.4. Correlation Analyses

All tested correlations are provided in the Supplementary Table S1; here we report only significant findings (*p* < 0.05).

#### 3.4.1. COPD

In COPD patients, significant correlations (*p* < 0.05) were observed between oxygen desaturation after 6MWT and endothelin-1 (ρ = −0.32, *p* = 0.018), as well as between oxygen saturation and lung volume on CT (ρ = 0.28, *p* = 0.041). Borg dyspnea scores were also positively correlated with endothelin-1 (ρ = 0.30, *p* = 0.026). These findings suggest that endothelin-1 may act as a mediator linking hypoxemia, subjective dyspnea, and structural lung alterations, consistent with endothelin pathway activation under hypoxic and inflammatory stress.

#### 3.4.2. SSc-ILD

In SSc-ILD, dyspnea scores correlated negatively with eGFR (ρ = −0.26 to −0.38, *p* < 0.05), while oxygen saturation correlated inversely with endothelin-1 (ρ = −0.29, *p* = 0.027), pointing to pulmo-renal interactions. Unlike COPD, where endothelin-1 predominated, SSc-ILD associations reflected systemic fibrotic remodeling and multi-organ vasculopathy.

Overall, the correlation analysis highlights context-specific cardio-pulmo-renal interactions: endothelin-driven hypoxia-linked mechanisms in COPD, and vascular–fibrotic involvement with renal participation in SSc-ILD.

## 4. Discussion

In this one-year longitudinal study with two assessment points, all patients had a normal baseline eGFR: 90.37 mL/min/1.73 m^2^ in COPD and 92.4 mL/min/1.73 m^2^ in SSc-ILD. Across both groups, eGFR declined by 6.76% in COPD and 3.16% in SSc-ILD, while the albumin-to-creatinine ratio (ACR) increased by 21% in COPD and by 6.7% in SSc-ILD. In both groups, median ACR values remained within KDIGO category A1 (<30 mg/g), and the modest 12-month increases (+21% in COPD, +6.7% in SSc-ILD) were non-significant, suggesting a trend rather than clinically relevant change.

A 6.76% decrease in eGFR can be interpreted in two ways. Biologically, it may seem modest; however, according to KDIGO (2012), a sustained annual decline ≥5 mL/min/1.73 m^2^ is considered rapid CKD progression [25]. Importantly, the same absolute reduction translates into different relative changes depending on baseline eGFR: for instance, a 5 mL/min decline represents approximately 5.6% at a baseline of 90 mL/min/1.73 m^2^ but ~8.3% if the baseline is 60 mL/min/1.73 m^2^. Therefore, expressing changes both in absolute and relative terms allows for a more consistent comparison across populations with a different baseline kidney function. We enrolled patients without CKD at baseline and observed a downward trend in eGFR and an upward trend in ACR over time. Thus, it is reasonable to hypothesize that a subset of patients may develop CKD, warranting continued follow-up to capture year-to-year fluctuations in eGFR. This interpretation is consistent with a systematic review and meta-analysis showing that COPD substantially increases the risk of incident CKD, and that CKD, in turn, significantly elevates mortality in COPD [4]. Acute COPD exacerbations may precipitate CKD: inflammatory mediators (e.g., TNF-α, IL-6, C-reactive protein) can impair renal function by acting on tubular cells and glomeruli, initiating inflammation-mediated apoptosis and fibrotic processes in the kidneys [26,27]. Oxidative stress related to pulmonary pathology has also been proposed to induce microvascular apoptotic injury and renal endothelial dysfunction, thereby reducing renal perfusion [28,29,30].

Median endothelin-1 across the two time points was higher in COPD (47.1 pg/mL) than in SSc-ILD (38.9 pg/mL). Levels of endothelin-1 increased both statistically and biologically in both groups; notably, the magnitude of increase in COPD was 3.5-fold greater. Endothelin-1 is essential for vascular tone’s regulation and is a potent vasoconstrictor. Its level rises in response to inflammatory cytokines, correlates with lung function impairment in COPD, and contributes to pulmonary hypertension. Even in stable COPD, persistent endothelial dysfunction, chronic hypoxia, oxidative stress, and systemic inflammation—often linked to smoking—drive a more pronounced increase in ET-1. In SSc-ILD, ET-1 elevation reflects slower fibrotic remodeling and vascular changes, consistent with more modest one-year dynamics. In SSc-ILD, anti–angiotensin II type 1 receptor and anti–endothelin-1 type A receptor antibodies, together with activation of the renin–angiotensin and endothelin systems, promote fibrosis progression, vasoconstriction, and vascular injury—mechanisms underlying the increase in endothelin-1 [3]. What remains unclear is why the rise in endothelin-1 in COPD was 3.5 times greater than in SSc-ILD despite lower baseline values. One plausible explanation is activation of pulmo-renal mechanisms as an adaptive response to chronic inflammation and hypoxia. This is supported by the fact that the median FEV_1_ (68.1% predicted) and FVC (42% predicted) were reduced in COPD and further declined by 4% and 7%, respectively.

Median galectin-3 across the two time points was higher in the SSc-ILD group. A statistically (*p* = 0.043) and biologically (10.2%) significant increase in galectin-3 occurred only in ILD, likely reflecting pulmonary fibrosis more strongly. Gal-3 dynamics differed between groups, with higher levels in SSc-ILD reflecting systemic fibrotic remodeling, whereas stable COPD showed weaker changes due to predominance of chronic airway inflammation. Galectin-3 plays a central role in fibrotic remodeling of the lungs, heart, and kidneys and participates in immune cell activation and tissue remodeling [31,32,33]. Faludi et al. reported that galectin-3 >10.25 ng/mL was the best predictor of all-cause mortality in SSc [34]. Several studies indicate that galectin-3 is directly associated with multiple pulmonary arterial hypertension phenotypes. Emre et al. found elevated galectin-3 in idiopathic pulmonary fibrosis versus healthy controls, with levels decreasing under antifibrotic therapy [35]; the decline correlated positively with the rate of FVC reduction [36]. Collectively, these data suggest galectin-3 is a key driver of fibrosis, including systemic fibrosis. However, experimental mouse studies showed that galectin-3 can both protect renal tubules from chronic injury by limiting apoptosis and, conversely, enhance matrix remodeling and attenuate fibrosis [37]. Autopsy data in SSc revealed that over 50% of renal lesions reflected nonspecific inflammatory changes unrelated to scleroderma renal crisis, and 45% represented multi-organ involvement [38]. Therefore, the clinical meaning of the observed rise in galectin-3 in our cohort remains uncertain and requires further longitudinal assessment with outcome-oriented endpoints.

The increase in galectin-3 among SSc-ILD patients is consistent with chest CT morphometrics. Median total lung volume over two time points in ILD was 3254.8 mL—at the lower limit of normal and half that in COPD (6831.3 mL). Over one year, lung volumes declined by 6.08% in ILD. CT density measurements also demonstrated lower mean attenuation values in ILD, trending toward −780 HU, compared to −880 HU in COPD. In slice L6, SSc-ILD patients showed a statistically significant 0.13% increase in lung tissue density. COPD patients exhibited heterogeneous CT changes: R1 (+0.07%), L1 (−0.21%), R2 (−0.01%)—indicating rarefaction—and R4 (−0.35%)—indicating consolidation. Saldana et al. found that CT density metrics correlated with physiologic impairment and visual CT scoring in SSc-ILD, although prognostic effects were inconsistent [39]. Conversely, higher densitometric lung density has been linked to poorer survival in IPF. Quantitative CT-derived parameters were comparable to clinical and functional measures [39].

In summary, COPD patients demonstrated a greater increase in endothelin-1 (+83.78%) against a background of reduced FEV_1_ and FVC, a 6.7% decline in eGFR, and a 21% rise in ACR. This pattern may reflect inflammation- and hypoxia-driven activation of endothelin-1 and the renin–angiotensin–aldosterone system, leading to significant vasoconstriction, pulmonary hypertension, and eGFR decline with evolving CKD. Further clinical studies are warranted to characterize these pulmo-renal interactions, the rates of functional decline in both systems, and their impact on outcomes.

Conversely, SSc-ILD patients showed a 6.08% annual reduction in lung volume (already low at baseline), lower CT densitometry values across slices, and an increase in galectin-3—suggestive of progressive pulmonary fibrosis rather than renal involvement. This may relate to the pleiotropic, context-dependent actions of galectin-3 or to relatively slow rates of multi-organ progression. Future studies should examine the kinetics and spatial distribution of CT density changes in relation to lung function, other organ systems, and renal outcomes.

This study has several limitations: it is cohort-based, one year in duration, with a limited sample of COPD and ILD patients. eGFR was estimated indirectly using the CKD-EPI creatinine-based equation (2021), which may be imprecise.

### Limitations

This study has several limitations. Although the sample size was calculated a priori to achieve adequate statistical power, its single-center nature and moderate cohort size (n = 112) limit the generalizability of the findings to other regions and ethnic groups. The design included one year of screening clinically stable patients and a subsequent 12-month follow-up with two assessment points, which does not capture long-term trajectories beyond this interval. Preliminary clinical data from the screening year were used to exclude patients with exacerbations, but they were not incorporated into the quantitative modeling of trajectories. All participants received pharmacotherapy under real-world conditions; while this reduces the likelihood of acute treatment effects, the non-standardized regimens and their possible adjustments could have influenced ET-1/Gal-3 levels and CT metrics, potentially attenuating the observed changes. The inclusion and exclusion criteria (age 25–65 years; exclusion of baseline eGFR < 60 mL/min/1.73 m^2^ and significant comorbidities; exclusion of patients with combined COPD and ILD) further narrow the external validity but were necessary to minimize the impact of non-pulmonary factors on the observed dynamics. Future multicenter studies with a longer follow-up and adjustment for pharmacotherapy are warranted to confirm and extend these findings.

At the same time, this study has several strengths. The prospective two-stage design (a screening year to include only stable patients followed by a one-year follow-up) minimized inter-individual variability and allowed reliable within-group comparisons. Comprehensive characterization of each participant using harmonized biomarkers (ET-1, Gal-3), standardized eGFR assessment, and quantitative HRCT densitometry provided an integrated view of vascular, renal, and pulmonary alterations. The inclusion of two clinical groups (COPD and SSc-ILD) enabled direct comparison of pathophysiological trajectories, while conducting the study under real-world pharmacotherapy conditions increases the clinical relevance of the results.

Patients in both groups were not restricted in pharmacotherapy; thus, drug effects could not be disentangled. Only patients with established diagnoses were enrolled; therefore, trajectories from onset of disease remain unknown. Given the short observation window, we interpreted our results cautiously to avoid oversimplified conclusions.

## 5. Conclusions

We observed several unexpected findings: endothelin-1 and eGFR changed more markedly in COPD than in SSc-ILD. Although both groups exhibited rising endothelin-1 and declining eGFR, the increase in endothelin-1 was 3.5-fold greater and the eGFR decline twice as large in COPD compared with SSc-ILD. This pattern likely reflects systemic inflammation and endothelial dysfunction rather than fibrosis progression, supporting the pulmo-renal continuum highlighted in this study.

In SSc-ILD, the observed changes mainly indicated pulmonary fibrosis progression, as reflected by a 6% annual reduction in lung volume and densitometric consolidation on CT slices (L6). Notably, correlations between endothelin-1 and desaturation/dyspnea in COPD, and between dyspnea and eGFR in SSc-ILD, further support disease-specific pulmo-renal interactions. Given the systemic nature of SSc-ILD, similar fibrotic processes may occur in other organs. Future research should explore renal and multi-organ fibrosis to better understand pulmo-renal relationships.

## Figures and Tables

**Table 1:** Methodology for selecting axial slices for chest CT-based lung densitometry.

Slice	Anatomical Landmark (Right Lung)	Slice	Anatomical Landmark (Left Lung)
R1	Aortic arch—right side	L1	Aortic arch—left side
R2	1 cm below the carina—right side	L2	1 cm below the carina—left side
R3	At the level of pulmonary vein entry—right	L3	At the level of pulmonary vein entry—left
R4	Midpoint between R3 and R5—right side	L4	Midpoint between L3 and L5—left side
R5	2 cm above the hemidiaphragm—right side	L5	2 cm above the hemidiaphragm—left side
R6	1 cm below the hemidiaphragm—right side	L6	1 cm below the hemidiaphragm—left side

**Table 2:** Baseline demographic and clinical characteristics of the study population.

Variable	COPD 58 n (%)	SSc-ILD 54 n (%)
Gender		
Male	50 (86.3%)	12 (22.2%)
Female	8 (13.7%)	42 (77.8%)
	Median [QR]	Median [QR]
Age	56.0 [52.0–60.0]	53 [49.0–57.0]
Smoking status	58 (100%)	3 (5.5%)
Spirometry Median [QR]		
FEV1% predicted	69.1 [61.0–76.0]	72.5 [66.0–80.0]
FVC% predicted	69.7 [64.0–76.5]	77.5 [71.0–84.0]

**Table 3:** One-year log-transformed change (log_2_FC) of median values and corresponding percentage change (Δ%) in COPD and SSc-ILD cohorts.

Marker (Unit)	COPD				SSc-ILD			
	Median Δ (2024–2023)	Median_log_2_FC	*p*	Δ%	Median Δ (2024–2023)	Median_log_2_FC	*p*	Δ%
GFR (mL/min/1.73 m^2^)	90.37	−0.101	0.001 *	−6.76	92.4	−0.046	0.029 *	−3.16
Endothelin-1 (pg/mL)	38.9	0.878	0.0002 *	83.78 **	47.1	0.308	0.0001 *	23.83 **
Galectin-3 (ng mL)	16.3	0.018	0.964	1.26	21.3	0.140	0.043	10.20
FVC (% predicted)	68.1	−0.059	0.01 *	−4.01	78.25	0.041	0.326	2.90
ACR (mg g)	3.05	0.275	0.281	21.00 **	4.65	0.092	0.681	6.58
FEV_1_ (% predicted)	42	−0.103	0.584	−6.89	73.8	0.068	0.128	4.86
Total lung volume (cm^3^)	683.3	0.042	0.146	2.95	3254.8	−0.091	0.020 *	−6.08
R1 density (HU)	−877	0.001	0.001 *	0.07	−800.8	−0.007	0.109	−0.50
L1 density (HU)	−870.8	0.003	0.004 *	0.21	−797.5	0.002	0.92	0.13
R2 density (HU)	−874.5	0.0001	0.002 *	0.01	−788.5	−0.007	0.651	−0.50
L2 density (HU)	−871.8	0.001	0.414	0.07	−801	0.001	0.484	0.06
R3 density (HU)	−877.5	−0.003	0.13	−0.21	−787.3	0.003	0.565	0.19
L3 density (HU)	−876.3	0.007	0.95	0.49	−797.3	0.009	0.726	0.69
R4 density (HU)	−879.5	−0.005	0.016 *	−0.35	−785.5	−0.02	0.505	−1.39
L4 density (HU)	−885.5	−0.006	0.354	−0.42	−785	−0.009	0.321	−0.64
R5 density (HU)	−884	−0.011	0.113	−0.76	−784.5	0.003	0.908	0.19
L5 density (HU)	−888.5	−0.002	0.694	−0.14	−780.5	0.004	0.353	0.32
R6 density (HU)	−895	−0.003	0.564	−0.21	−782.75	−0.01	0.108	−0.70
L6 density (HU)	−895.3	−0.003	0.117	−0.21	−788.5	−0.002	0.004 *	−0.13
SpO_2_ after 6MWT (%)	0	−0.015	0.128	−1.058	0	−0.023	0.015 *	−1.604
Borg scale after 6MWT (score)	0	0.193	0.671	14.286	0	0.415	0.002 *	33.333

* Statistically significant (Wilcoxon test) *p* < 0.05; ** biologically significant |log_2_FC| > 0.20.

**Table 4:** Comparison of median changes (Δ) in study parameters between the COPD and SSc-ILD groups. The table reports the median change in each group, the between-group difference (Δdiff), the corresponding Mann–Whitney U *p*-value, and sample sizes.

Marker (unit)	Median Δ (COPD)	Median Δ (SSc-ILD)	Δ Difference (SSc-ILD − COPD)	*p*-Value (Mann–Whitney U)
GFR (mL/min/1.73 m^2^)	−3.3	−2.65	0.6	0.628
Endothelin-1 (pg/mL)	10.722	4.7231	−5.9 **	0.447
Galectin-3 (ng mL)	−0.25	1.3	1.6 **	0.101
FVC (% predicted)	−4	1.05	5.1 **	0.005 *
ACR (mg g)	0	0	0	0.396
FEV_1_ (% predicted)	1.5	2	0.5	0.358
Total lung volume (cm^3^)	−93	−117.5	−24.5 **	0.718
R1 density (HU)	−2	−2	0	0.355
L1 density (HU)	−2.5	−1	1.5 **	0.075
R2 density (HU)	−2	−1	1	0.028 *
L2 density (HU)	1	−1	−2 **	0.256
R3 density (HU)	2	0	−2 **	0.691
L3 density (HU)	0.5	−0.5	−1	0.937
R4 density (HU)	2	0.5	−1.5 **	0.407
L4 density (HU)	1.5	2	0.5	0.739
R5 density (HU)	2	0	−2 **	0.398
L5 density (HU)	−1	0.5	1.5 **	0.353
R6 density (HU)	1	3	2 **	0.217
L6 density (HU)	2.5	2.5	0	0.116
SpO_2_ after 6MWT (%)	0.0	0.0	0.0	0.575
Borg scale after 6MWT (score)	0.0	0.0	0.0	0.018 *

Statistically significant: * *p* < 0.05 (Mann–Whitney U test); biologically significant: ** Δdiff > 1 unit.

## Data Availability

All data supporting the findings of this study, as well as the R scripts used for data processing and analysis, are available in the Supplementary Materials. Additional information is available from the corresponding author upon reasonable request.

## Supplementary Materials

The following supporting information can be downloaded at: https://www.mdpi.com/article/10.3390/medicina61091572/s1, File S1: dataset.xlsx; File S2: analysis_code.R. Table S1: Spearman correlation coefficients (ρ) between clinical, functional, and biomarker parameters in COPD and SSc-ILD.

## Abbreviations

The following abbreviations are used in this manuscript:

ACR	Albumin-to-creatinine ratio
CKD	Chronic kidney disease
CLD	Chronic lung disease
COPD	Chronic obstructive pulmonary disease
CT	Computed tomography
eGFR	Estimated glomerular filtration rate
ELISA	Enzyme-linked immunosorbent assay
ET-1	Endothelin-1
FEV_1_	Forced expiratory volume in one second
FVC	Forced vital capacity
Gal-3	Galectin-3
GOLD	Global Initiative for Chronic Obstructive Lung Disease
HRCT	High-resolution computed tomography
HU	Hounsfield units
ILD	Interstitial lung disease
SSc-ILD	Systemic sclerosis-associated interstitial lung disease
