# Supplementary material for: Assessing Fibrosis Progression and Endothelial Dysfunction in SSc-ILD and COPD: An Integrated Biomarker and CT Densitometry Approach
Source: Medicina (Kaunas). 2025 Aug 31;61(9):1572. doi: 10.3390/medicina61091572 (PMC12471967; doi:10.3390/medicina61091572)
Supplement: Supplementary file 1 [file medicina-61-01572-s001.zip › analysis_code_R.pdf]

```r
# R Script for Data Analysis
# Works with dataset where variables have format *_23 and *_24 (e.g., GFR_23, GFR_24)

# --- Step 1. Load packages ---
library(readxl)
library(dplyr)
library(ggplot2)
library(ggrepel)

# --- Step 2. Import data from Excel ---
# replace "Dataset.xlsx" with your actual file name
df <- read_excel("Dataset.xlsx")

# --- Step 3. Collect variable names ---
markers <- sub("_23$", "", grep("_23$", names(df), value = TRUE))

# --- Step 4. Calculate log2FC and p-value ---
results <- lapply(markers, function(m) {
  v23 <- df[[paste0(m, "_23")]]
  v24 <- df[[paste0(m, "_24")]]

  log2FC <- log2(median(v24, na.rm = TRUE) / median(v23, na.rm = TRUE))
  p_value <- wilcox.test(v24, v23, paired = TRUE)$p.value

  data.frame(
    marker = m,
    log2FC = log2FC,
    p_value = p_value,
    neglog10p = -log10(p_value)
  )
}) %>% bind_rows()
```

```r
# --- Step 5. Add labels and significance ---
label_map <- c(
  endothelin1 = "ET-1",
  galectin = "Gal-3",
  GFR = "GFR",
  FVC = "FVC",
  ACR = "ACR",
  Vol = "Vol",
  SpO2 = "SpO2",
  Borg = "Borg",
  OFV1 = "FEV1"
)
results$marker_label <- ifelse(results$marker %in% names(label_map),
                               label_map[results$marker],
                               results$marker)

results <- results %>%
  mutate(significant = ifelse(p_value < 0.05, "yes", "no"))

# --- Step 6. Volcano plot ---
volcano_plot <- ggplot(results, aes(x = log2FC, y = neglog10p)) +
  geom_point(shape = 4, aes(color = significant == "yes"), size = 2) +
  geom_vline(xintercept = 0, linetype = "dashed", color = "black") +
  geom_vline(xintercept = c(-0.2, 0.2), linetype = "dashed", color = "black") +
  geom_hline(yintercept = -log10(0.05), linetype = "dashed", color = "blue") +
  geom_text_repel(
    data = subset(results, p_value < 0.05 | abs(log2FC) > 0.2),
    aes(label = marker_label), size = 3
  ) +
  scale_color_manual(values = c("TRUE" = "red", "FALSE" = "grey40")) +
  labs(
    x = expression("ME log"[2]*"FC (Me 2024 / Me 2023)"),
```

```
  y = expression(-log[10]*"(p-value) (Wilcoxon)")
 ) +
 coord_cartesian(ylim = c(0, 4)) +
 theme_minimal() +
 theme(
  legend.position = "none",
  axis.title = element_text(size = 12),
  axis.text = element_text(size = 10)
 )

# --- Step 7. Save figure ---
ggsave("volcano_plot.png", plot = volcano_plot, width = 7, height = 5, dpi = 300)

# --- Step 8. Save results table ---
write.csv(results, "results_log2FC.csv", row.names = FALSE)
```
