# Supplementary material for: Assessing Fibrosis Progression and Endothelial Dysfunction in SSc-ILD and COPD: An Integrated Biomarker and CT Densitometry Approach
Source: Medicina (Kaunas). 2025 Aug 31;61(9):1572. doi: 10.3390/medicina61091572 (PMC12471967; doi:10.3390/medicina61091572)
Supplement: Supplementary file 1 [file medicina-61-01572-s001.zip › Spearman_table.pdf]

Table S1. Spearman correlation coefficients (ϱ) between clinical, functional, and biomarker parameters in COPD and SSc-ILD.

Group	Target	Marker	rho	p
COPD	Borgskale_after_6MWT_23	L4_23	-0,23617	0,085
COPD	Borgskale_after_6MWT_23	L4_24	-0,25337	0,064
COPD	Borgskale_after_6MWT_23	R6_23	-0,23044	0,093
COPD	Borgskale_after_6MWT_23	endothelin1_24	0,302952	0,025
COPD	Borgskale_after_6MWT_23	endothelin1_24	0,254055	0,063
COPD	SaO2%_after_6MWT_23	ACR_23	-0,22866	0,096
COPD	SaO2%_after_6MWT_23	endothelin1_24	-0,32103	0,017
COPD	SaO2%_after_6MWT_24	L2_24	-0,23769	0,083
COPD	SaO2%_after_6MWT_24	R1_23	-0,22571	0,100
COPD	SaO2%_after_6MWT_24	R1_24	-0,23717	0,084
COPD	SaO2%_after_6MWT_24	R2_24	-0,24013	0,080
COPD	SaO2%_after_6MWT_24	Vol_24	0,279691	0,040
COPD	SaO2%_after_6MWT_24	endothelin1_24	-0,28674	0,035
SSc-ILD	Borgskale_after_6MWT_23	GFR_24	-0,26407	0,045
SSc-ILD	Borgskale_after_6MWT_24	GFR_23	-0,26117	0,047
SSc-ILD	Borgskale_after_6MWT_24	GFR_24	-0,38336	0,002
SSc-ILD	Borgskale_after_6MWT_24	OFV1_24	0,334525	0,010
SSc-ILD	SaO2%_after_6MWT_23	ACR_24	-0,21822	0,099
SSc-ILD	SaO2%_after_6MWT_23	endothelin1_24	-0,29073	0,026
